# Biogenic silver/silver chloride nanoparticles inhibit human cancer cells proliferation in vitro and Ehrlich ascites carcinoma cells growth in vivo

**DOI:** 10.1038/s41598-022-12974-z

**Published:** 2022-05-26

**Authors:** Syed Rashel Kabir, Farhadul Islam, A. K. M. Asaduzzaman

**Affiliations:** Department of Biochemistry and Molecular Biology, Faculty of Science, University of Rajshahi, Rajshahi, 6205 Bangladesh

**Keywords:** Biochemistry, Cell biology, Molecular biology, Stem cells

## Abstract

Silver/silver chloride nanoparticles (Ag/AgCl-NPs) were synthesized for the first time from the herbal *Geodorum densiflorum* rhizome extracts and characterized by different techniques. The surface plasmon resonance peak at 455 nm was observed in the UV–Visible spectrum, the average particle size of 25 nm was determined by SEM, XRD reflection peaks (28.00°, 32.42°, 38.28°, 46.38°, 54.94°, 57.60°, 64.64°, and 67.48°) indicated the presence of Ag-NPs and AgCl-NPs, heat stability was confirmed by TGA and FTIR analysis indicated the presence of alcohol/phenol, alkanes, primary amines, nitro compounds, alkyl chloride functional groups. The synthesized Ag/AgCl-NPs, previously synthesized *Kaempferia rotunda* and *Zizyphus mauritiana* mediated Ag/AgCl-NPs separately inhibited the proliferation of BxPC-3 cells with the IC_50_ values of 7.8, 17.1, and 20.1 µg/ml, respectively. In the case of MCF-7 cells, the IC_50_ values of *G. densiflorum*- Ag/AgCl-NPs and *K. rotunda-*Ag/AgCl-NPs were 21.5 and 23.5 µg/ml, respectively. Whereas the IC_50_ of *G. densiflorum*-Ag/AgCl-NPs was 28.0 µg/ml against glioblastoma stem cells (GSCs). Induction of apoptosis in GSCs, BxPC-3 and MCF-7 cells was noted followed by NPs treatment. In GSCs, the expression level of *NFκB, TNFα, p21*, and *TLR9* genes were upregulated after treatment with *G. densiflorum*-Ag/AgCl-NPs while in the MCF-7 cells, the expression of *p53, FAS, Caspase-8* and *-9, NFκB, MAPK, JNK* and *p21* genes were increased. *G. densiflorum*-Ag/AgCl-NPs inhibited 60% and 95% of EAC cells growth at the doses of 2 and 4 mg/Kg/day after intraperitoneal treatment with five consequent days, respectively. A remarkable improvement of hematological parameters with the decreased average tumor weight and increase of 75% life span of *G. densiflorum*-Ag/AgCl-NPs treated mice were observed. Altogether, this study reported for the first time in vitro anticancer activity of biogenic *G. densiflorum*-Ag/AgCl-NPs against GSC cells along with MCF-7 and BxPC-3 cells and in vivo anticancer properties against EAC cells.

## Introduction

One of the emerging fields of nanotechnology is nanomedicine. Recently, nanomedicine is widely used in the development of antimicrobial agents and various drugs for the treatment of human diseases, including cancer^1–4^. One of the well-known candidates for nanomedicine is nanoparticles. Different Nobel metals e.g. platinum, gold, and silver are used for the synthesis of nanoparticles using chemical, biological, electrochemical, and photochemical methods. Among them, silver metal and biological methods gained lots of interest among the researchers. Thus, researchers are trying to synthesize silver/silver chloride nanoparticles (Ag/AgCl-NPs) using a biological method where microorganisms or extracts of the various parts of plants e.g., roots, fruits, bark, leaves, are used. These extracts contained proteins, terpenoids, flavonoids, and cofactors that serve as capping or reducing agents of the silver^4–9^. Several articles reported that biogenic silver/silver chloride nanoparticles induced apoptosis in embryonic zebrafish and showed anticancer activity against different cancer cells both in vitro and in vivo in mice^1,2,10–17^. Interestingly, the toxicity of the biogenic silver is several times higher for cancer cells than that of normal cells^18^. However, the toxicity depends on the biological sources and synthesis parameters^19,20^. Researchers have been synthesizing silver/silver chloride nanoparticles to examine the anticancer activities against different cancer cells to find the most suitable anticancer agents. Recently, we synthesized silver/silver chloride nanoparticles from *Kaempferia rotunda* rhizome and *Zizyphus mauritiana* extracts and the anticancer activity was elucidated against different cancer cell lines^1,2^. Where *K. rotunda*-Ag/AgCl-NPs inhibited the Ehrlich ascites carcinoma (EAC) cells in mice and glioblastoma stem cells (GSCs) in vitro more efficiently than the *Z. mauritiana*-Ag/AgCl-NPs^1^.

*Geodorum densiflorum* rhizome, is a herb that is used for the preparation of traditional medicine in this subcontinent^19^. Recently, we purified a lectin from the rhizome of *G. densiflorum* that showed potent anticancer activity against EAC cells^21^. Since lectin from *G. densiflorum* exhibited significant anticancer activity at least against EAC cells, thus, it is hypothesized that biogenic NPs derived from *G. densiflorum* would have potential anticancer activity with a better host-toxic profile against other cellular models. Thus, the aims of the present works were the synthesis of *G. densiflorum*-Ag/AgCl-NPs followed by characterization by Scanning Electron Microscope (SEM), X-ray powder diffraction (XRD), Thermal Gravimetric Analysis (TGA), and Fourier-transform infrared spectroscopy (FTIR), respectively. Also, the comparative anticancer activity of *G. densiflorum-*Ag/AgCl-NPs, *K. rotunda-*Ag/AgCl-NPs and *Z. mauritiana*-Ag/AgCl-NPs were investigated against human pancreatic cancer cells (BxPC-3) for the first time. In addition, the underlying mechanism of anticancer activity *G. densiflorum-*Ag/AgCl-NPs against human GSCs and MCF-7 cells were elucidated in this study. Furthermore, cell proliferation, average tumor weight, hematological parameters and life span of EAC-bearing mice were examined for the first time in this study followed by *G. densiflorum-*Ag/AgCl-NPs treatment.

## Materials and method

### Chemicals and reagents

The chemicals which were used were purchased from world-leading companies e.g. Gibco, Promega, Amresco, Life technology, Applied biosystem, etc.

### Sample collection and preparation of extracts

The medicinal village of Natore District, Bangladesh is a commercial place where different kinds of medicinal herbs are cultivated and available for sale to individuals and companies. From the market, we bought fresh *G. densiflorum* rhizome. We declare that the collection of plant material is in accordance with relevant institutional, national and international guidelines and legislation. *G. densiflorum* rhizome was unsoiled and exocarp was removed and cut into small pieces. Then cleaned with deionized water and homogenized with ten volumes of 10 mM of Tris–HCl buffer. After centrifuging the homogenate at 10,000 g for 30 min at 4 °C, clear supernatant was obtained for silver nanoparticle synthesis.

### Synthesis of silver/silver chloride nanoparticles

For the synthesis of Ag/AgCl-NPs nanoparticles, AgNO_3_ solution was mixed with the clear *G. densiflorum* rhizome extract separately. When the AgNO_3_ concentration reached to 1.0, 2.0, and 4.0 mM then the mixture was incubated in sunlight. Approximately 2 h later the mixture’s color was changed from transparent to deep brown. Then the products were analyzed by UV–visible spectroscopy at the wavelength from 250 to 700 nm. Based on peak height, 4.0 mM AgNO_3_ was selected for the best synthesis. For the synthesis of nanoparticles on a large scale, 100 g of *G. densiflorum* rhizome was homogenized with 1 L of deionized water. After centrifuging at 10,000 g for 30 min at 4 °C around 1 L of clear supernatant was obtained. Finally, 1.0 M AgNO_3_ solution was added to the supernatant with mixed until the concentration reached 4.0 mM. Then the mixture was incubated for 2 h in the sunlight. After developing the deep brown color, the colloidal solution was placed in tubes and centrifuged at 10,000 rpm for about 30 min. Pellet obtained after centrifugation was dissolved with deionized water and repeated the centrifugal process three times. Finally, A minor portion of the colloidal sample was stored at 4 °C for biological activity assays and the rest of the colloidal sample was lyophilized for various characterizations. *Z. mauritiana* fruit extract-mediate and *K. rotunda* rhizome extract-mediate Ag/AgCl-NPs were synthesized according to the protocol previously published by Kabir et al.^1,2^.

### Characterization of *G. densiflorum*-Ag/AgCl-NPs

The shape and size of the nanoparticles were confirmed by JSM-7610F (JEOL, Japan) Scanning Electron Microscope. The crystalline form of the synthesized Ag/AgCl-NPs was determined by X-ray powder diffraction analysis with the help of CuK_α_1 radiation of Ultima IV (Rigaku, Japan) X-ray diffractometer operated at 40 kV and 40 mA at a 2θ angle pattern and scanned in the region of 10–70°. The QualX2.0 software was used for the analysis of the XRD data. For the detection of the thermal stability of the synthesized *G. densiflorum*-Ag/AgCl-NPs, a thermogravimetric analyzer (PerkinElmer STA 8000, USA) controlled the 20 °C/min heating rate was used. For FTIR spectroscopic measurements, potassium bromide (KBr) and lyophilized Ag/AgCl-NPs were mixed with the ratio of 100:1, and PerkinElmer, Spectrum 100 (USA) system was used. The frequency in the range of 4000–225 cm^−1^ was used for the measurement with a resolution of 1 cm^−1^.

### Cell culture

The human glioma stem cells-3 (GSCs) was established by the Kunming Institute of Zoology ^22,23^, The cells were collected from the Zhao XuDong laboratory, Kunming Institute of Zoology (KIZ). BxPC-3 and MCF-7 were recruited from American Type Culture Collection (ATCC). GSCs was cultured according to Wan Peng et al.^22^ and MCF-7 and BxPC-3 cells were cultured according to the protocol used by Kabir et al.^2^. These cells related all experiments were done at the said laboratory, of KIZ. For the treatment of GSCs with the synthesized AgCl-NPs, at first 6 or 96 wells of flats bottom cell culture plates were coated with Laminin-phosphate buffer saline (PBS) (1:100) and incubated at 37 °C for 2.0 h. Then PBS with unbound Laminin (Gibco) was removed from the cell culture petri-dish or culture plates. GSCs were added to the DMEM/F12 medium (Gibco) and quickly seeded in the cell culture petri-dish or plates and cells were cultured in a 5% CO_2_ incubator at 37 °C until cells reached 80–90% confluence.

### MTS assay for cytotoxicity study

Cytotoxicity was studied according to Kabir et al.^1,2^ by using MTS assay (Promega, USA). For the stem cell culture, complete DMEM/F12 medium was used. On the other hand, for the MCF-7 and BxPC-3 culture, DMEM medium (Gibco) with 10% FBS (Gibco) was used. Around 2.0 × 10^4^ GSCs and BxPC-3 cells, and 1.0 × 10^4^ MCF-7 cells were taken in the respective medium and seeded to each well of the 96 wells cell culture plate. After that, the plate was incubated in a 37 °C incubator with a flow of 5% CO_2_ for 24 h. Then the cells were treated with the *G. densiflorum*-Ag/AgCl-NPs, *K. rotunda*-Ag/AgCl-NPs, and *Z. mauritiana*-Ag/AgCl-NPs at the concentration of 8.0–32.0 µg/ml for 48 h.

### Identification of caspase-3 protein expression in GSCs by the immunofluorescence assay

To detect the expression level of caspase-3 in GSCs, 2 × 10^4^ cells were seeded in each well of a 96 wells plate then the cells were treated with 16 μg/ml of *G. densiflorum*-Ag/AgCl-NPs for 48 h. Then the expression level of caspase-3 protein was detected by fluorescence microscope followed by the treatment with 4% paraformaldehyde, 0.1% Triton X-100, phosphate buffer saline, 10% goat serum, 0.5% tween-20, and 1% BSA in PBS and finally, caspase-3 primary antibody and Cy3 goat-anti Rabbit IgG antibody (Life technology) followed by the protocol described by Kabir et al.^1^.

### Staining of MCF-7 and BxPC-3 cells with the FITC labeled annexin V/PI

2.0 × 10^4^ BxPC-3 cells and 1.0 × 10^4^ MCF-7 cells were seeded to each well of the 96 wells plate and incubated for 24 h. Then the cells were treated for 48 h with the *G. densiflorum-*Ag/AgCl-NPs and *K. rotunda-*Ag/AgCl-NPs at the concentration of 16 µg/ml as mentioned above. After that BxPC-3 and MCF-7 cells were stained with the FITC labeled annexin V/PI (ebioscience, USA), according to Kabir et al.^1^. Finally, the stained plate was analyzed using a fluorescence microscope (Olympus IX71) and pictures were captured under different conditions to evaluate the changes in the cancer cells as well as detection of apoptosis.

### Detection of ROS in MCF-7 cells

MCF-7 cells (1.0 × 10^4^/well) in the 96 well plate were incubated with *G. densiflorum-*Ag/AgCl-NPs and *K. rotunda-*Ag/AgCl-NPs for 48 h. The cells were washed and stained with 2′,7′-dichlorofluorescein-diacetate (DCFH-DA) as discussed by Kabir et al.^2^.

### Changes of genes expressions in GSCs and MCF-7 cells

16 × 10^4^ MCF-7 cells and 32 × 10^4^ GSCs were taken in the respective buffer and seeded in each well of a 6-well plate. Around 24 h later cells were incubated with *G. densiflorum*-AgCl-NPs (16.0 μg/ml) for 48 h. After that RNA was isolated, cDNA was synthesized and for the preparation of the reaction mixture of real-time PCR, 2xSYBR green master mix (Applied Biosystem) was used according to Kabir et al.^1^. A list of the primers (TsingKe Biological Technology, China) was presented in Table 1. The condition for real-time PCR was as follows, 50 °C (2 min) and 95 °C (2 min) for 40 cycles 95 °C for 15 s and 60 °C for 1 min. BioRad thermal cycler was used and the data were analyzed by the Double Delta CT method. The data were normalized using the 18S gene.

**Table 1:** List of Primers.

NFkB	F	CCAGTATCCCGGTCCAGCTAT
	R	CACGTCCAACTCACTCCAAGG
TNFα	F	ATTGCCGCAGAAAGTTCTACG
	R	GTCCAGTTTCGTCTTCAGCTC
18S	F	GTAACCCGTTGAACCCCATT
	R	CCATCCAATCGGTAGTAGCG
TLR9	F	CTGCCTTCCTACCCTGTGAG
	R	GGATGCGGTTGGAGGACAA
NOTCH2	F	CAACCGCAATGGAGGCTATG
	R	GCGAAGGCACAATCATCAATGTT
p53	F	GCCCAACAACACCAGCTCCT
	R	CCTGGGCATCCTTGAGTTCC
P21	F	TGCAACTACTACAGAAACTGCTG
	R	CAAAGTGGTCGGTAGCCACA
Caspase-8	F	ACACAGTCGAGTAGACTCTCAAA
	R	AGGAAGTGATGCTCGTTCAGA
FAS	F	CCCAGTCCTTCACTTCTATGTTC
	R	GTAGCACAGTTCAGTCTCGAC
Caspase-9	F	CTGTCTACGGCACAGATGGAT
	R	GGGACTCGTCTTCAGGGGAA
JNK	F	GGGTATGCCCAAGAGGACAGA
	R	GTGTTGGAAAAGTGCGCTGG
MAPK	F	CGGTGTCAATGGTTTGGTGC
	R	GACGATGTTGTCGTGGTCCA

### Experimental animals and ethical clearance

Swiss albino mice were produced at our university and were maintained in accordance with the guidelines and regulations of the Institution of Biological Sciences, University of Rajshahi, Bangladesh for the Care and Use of Laboratory Animals. Also, the use of a minimal number of animals and their suffering was minimized in the current study. All the in vivo experiments were performed according to protocols approved by the Institutional Animal, Medical Ethics, Bio-safety and Bio-security Committee (IAMEBBC) for Experimentations on Animals, Human, Microbes and Living Natural Sources (293(13)320-IAMEBBC/IBSc) Institute of Biological Sciences (IBSc), University of Rajshahi, Bangladesh and confirmed to ARRIVE guideline. A total of 36 Swiss albino mice (males, 8 weeks old, 22–25 g weight) were housed in cages (6 mice/cage) with free access to food and water. All animals were kept under a 12-h/12-h light/dark cycle (lights on at 6:00 a.m.). The mice were sacrificed in the current study using a two-step process as per the approved guideline. Firstly, the mice were rendered unconscious through inhaled anesthetic agent (Isoflurane) exposure. Subsequently, they were killed by cervical dislocation while the animals were fully unconscious. Isoflurane was administrated by drop method in a container with a tightly fitted lid.

### In vivo EAC cell growth inhibition

EAC cells propagated intraperitoneally in the ambient of our departmental laboratory biweekly. EAC cells were collected in saline from Swiss albino mice bearing 6 or 7 days old ascites tumors and then 1 × 10^6^ cells in 0.1 mL of saline were injected intraperitoneally to 18 Swiss albino mice and incubated at room temperature for tumor inoculation. 24 h later, the mice were randomly distributed into three groups (six mice per group) and *G. densiflorum*-AgCl-NPs were injected intraperitoneally into two groups at the doses of 2.0 and 4.0 mg/kg/day for five consecutive days. The rest of the group was used as a control. The mice sacrificed on the seventh day of the EAC inoculation and EAC cells were collected in normal saline. Finally, cells were counted by a light microscope and the percent of cell growth inhibition was calculated.

### Determination of average tumor weight and survival time

Twelve Swiss albino mice were treated with EAC cells as described above and after 24 h one group (six mice per group) was treated with 4.0 mg/kg/day of *G. densiflorum*-AgCl-NPs for ten consecutive days. The NPs untreated group was used as a control. The third group of mice without EAC and nanoparticles was used as normal mice. After treatment with *G. densiflorum*-AgCl-NPs, changes in the weight of each mouse were recorded daily up to 20 days of EAC cells inoculation and the host survival time was recorded. Finally, the mean survival time (in days) and the percent increase of life span were calculated as described by Kabir et al.^24^.

### Hematological parameters of normal, EAC inoculated and *G. densiflorum*-AgCl-NPs treated on EAC inoculated mice

Mice that were used for the determination of average tumor weight and survival time were also subjected to the study of hematological parameters. About 50 µl of blood was drawn from the tail of each mouse of the EAC bearing control, *G. densiflorum*-AgCl-NPs treated EAC bearing and normal mice on the 12th day of EAC cells inoculation. Finally, the percentage of hemoglobin, total WBC and total RBC were counted.

## Results

### Synthesis of *G. densiflorum*-Ag/AgCl-NPs and the morphological characterization

**Figure 1:**
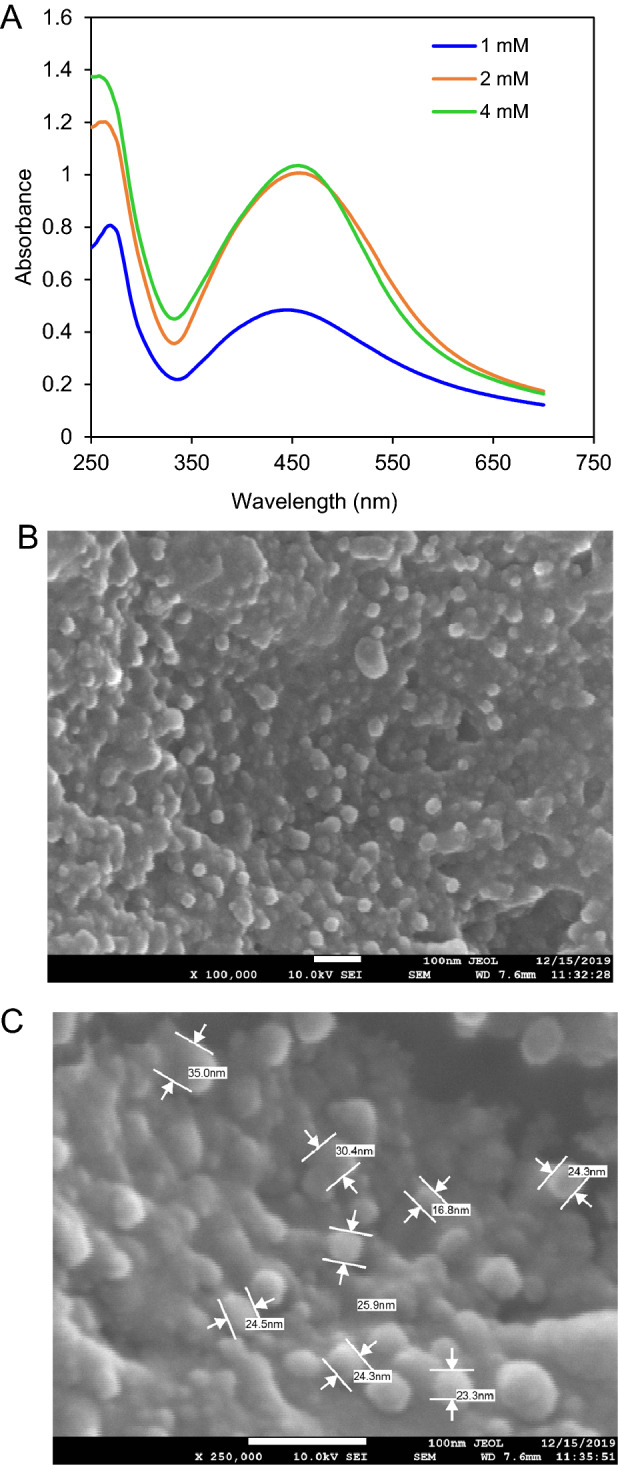
Biosynthesis and characterization of *G. densiflorum*-Ag/AgCl-NPs (**A**) UV–visible spectra indicating synthesis of nanoparticles after mixing different concentrations of AgNO_3_ with the rhizome extract. (**B**) SEM micrograph of the synthesized nanoparticles. (**C**) Morphology and size of the nanoparticles.

The deepest brown color solution was obtained after incubation with 4.0 mM of silver nitrate that preliminary supported the formation of *G. densiflorum-*Ag/AgCl-NPs. The maximum absorbance peak in the UV–visible spectra was observed near 455 nm (Fig. 1A). About 215 mg (15 mg/ml) of Ag/AgCl-NPs was obtained from 100 g of *G. densiflorum* rhizome. The picture of SEM indicated the synthesized Ag/AgCl-NPs were spherical and the average size was 25 nm (Fig. 1B and C).

### Structural, thermal and functional characterization of *G. densiflorum*-Ag/AgCl-NPs

**Figure 2:**
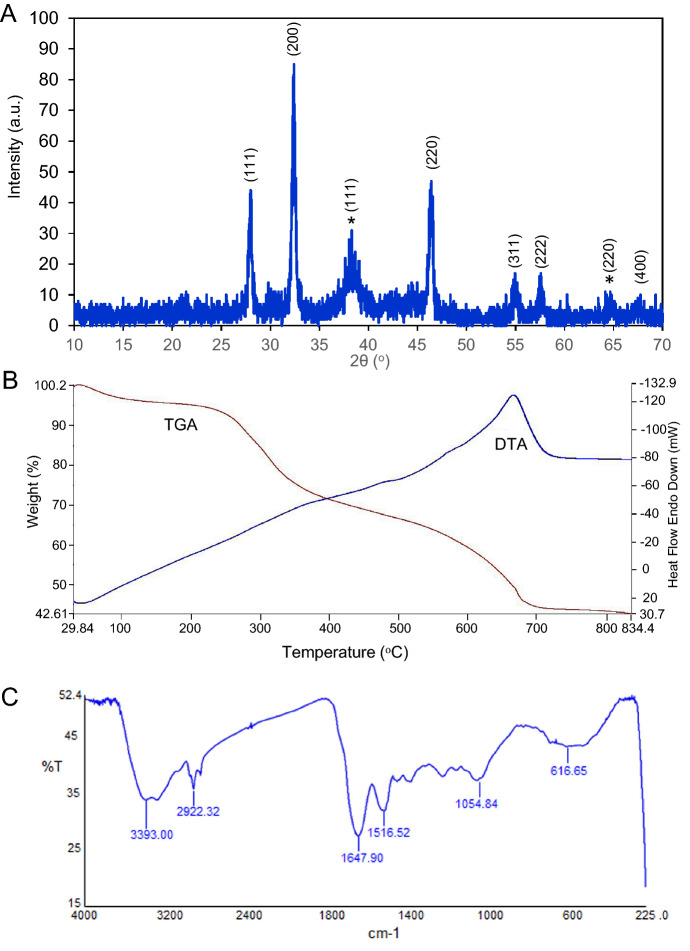
XRD, TGA and FTIR spectrum. (**A**) XRD pattern (**B**) TGA and (**C**) FTIR spectrum of the synthesized nanoparticles.

Formation of *G. densiflorum*-AgCl-NPs was specified from the XRD reflection peaks where 28.00°, 32.42°, 46.38°, 54.94°, 57.60° and 67.48° confirming the crystallographic planes [111], [200], [220], [311], [222], and [400], respectively [card no. 00-901-1666]. Whereas formation of AgNPs was confirmed from the reflection peaks of 38.28° and 64.64°, confirming the crystallographic planes [111], and [220], respectively [card no. 00-150-9146] (Fig. 2A). Crystal in the cubic form was confirmed for both cases. Also, TGA plot/profile confirmed the three steps weight loss of the synthesized Ag/AgCl-NPs at the temperature ranges from 30 to 100 °C; 100.1–350.0 °C and 350.1–675.0 °C, corresponding to 2.73%, 21.49%, 27.98% weight loss as shown in the Fig. 2B. Six major peaks (616.65, 1054.84, 1516.52, 1647.90, 2922.32 and 3393.00 cm^−1^) were obtained in the FTIR spectra of the synthesized *G. densiflorum*-Ag/AgCl-NPs as mentioned in Fig. 2C.

### Antiproliferative activity studied by MTS assay

**Figure 3:**
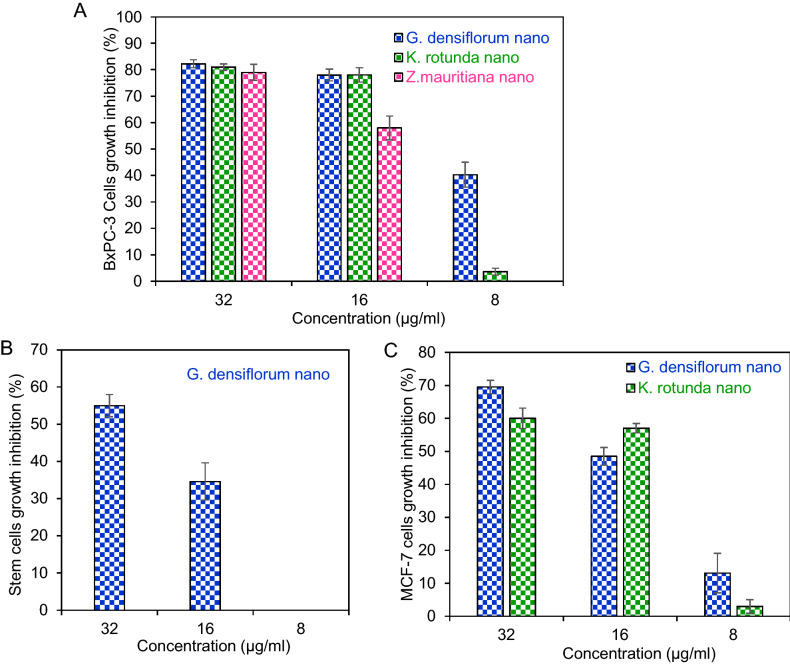
Antiproliferative activity against BxPC-3, GSCs and MCF-7. (**A**) Antiproliferative activity of *G. densiflorum*-Ag/AgCl-NPs, *K. rotunda*-Ag/AgCl-NPs and *Z. mauritiana*-Ag/AgCl-NPs against the BxPC-3. (**B**) Antiproliferative activity of *G. densiflorum*-Ag/AgCl-NPs against the GSCs. (**C**) Antiproliferative activity of *G. densiflorum*-Ag/AgCl-NPs and *K. rotunda*-Ag/AgCl-NPs against the MCF-7 cells. After incubation of the cells with different concentrations of Ag/AgCl-NPs, the inhibition ratio was calculated by Microsoft excel software (n = 3, mean ± S.D.).

Antiproliferative activity of *Z. mauritiana-*Ag/AgCl-NPs, *K. rotunda-*Ag/AgCl-NPs, and *G. densiflorum*-Ag/AgCl-NPs was evaluated against BxPC-3 cells. Around 80% cell growth inhibition was observed for the three nanoparticles at the concentration of 32 µg/ml. When the concentration was reduced to half, then a remarkable decrease of growth inhibition was observed only for the *Z. mauritiana*-Ag/AgCl-NPs. Finally, at 8 µg/ml concentration, no inhibition was observed for *Z. mauritiana*-Ag/AgCl-NPs, while 40.3% and 3.6% cell growth inhibition were observed for *G. densiflorum-* Ag/AgCl-NPs and *K. rotunda-*Ag/AgCl-NPs, respectively (Fig. 3A). IC_50_ values for *G. densiflorum*-Ag/AgCl-NPs, *K. rotunda-*Ag/AgCl-NPs and *Z. mauritiana-*Ag/AgCl-NPs were 7.8, 17.1 and 20.1 µg/ml, respectively. A 55% and 34.6% of GSCs growth were inhibited at the 32 and 16 µg/ml of *G. densiflorum*-Ag/AgCl-NPs, respectively. When the concentration was decreased to 8 µg/ml, no inhibition was observed (Fig. 3B). The IC_50_ was estimated to be 28.0 µg/ml. Growth inhibition of *K. rotunda-*Ag/AgCl-NPs and *G. densiflorum*-Ag/AgCl-NPs against MCF-7 cells were checked. 69.5%, 48.5% and 13.1% cells growth inhibition were observed for *G. densiflorum*-Ag/AgCl-NPs, at the concentration of 32, 16 and 8 µg/ml, respectively, while *K. rotunda*-Ag/AgCl-NPs inhibited 60%, 57%, and 3% of MCF-7 cells growth at the 32, 16 and 8 µg/ml, as showed in the Fig. 3C. The IC_50_ values were 21.5 and 23.5 µg/ml for *G. densiflorum-*Ag/AgCl-NPs, and *K. rotunda-*Ag/AgCl-NPs, respectively.

### Fluorometric studies of the treated GSCs, MCF-7, and BxPC-3 cells

**Figure 4:**
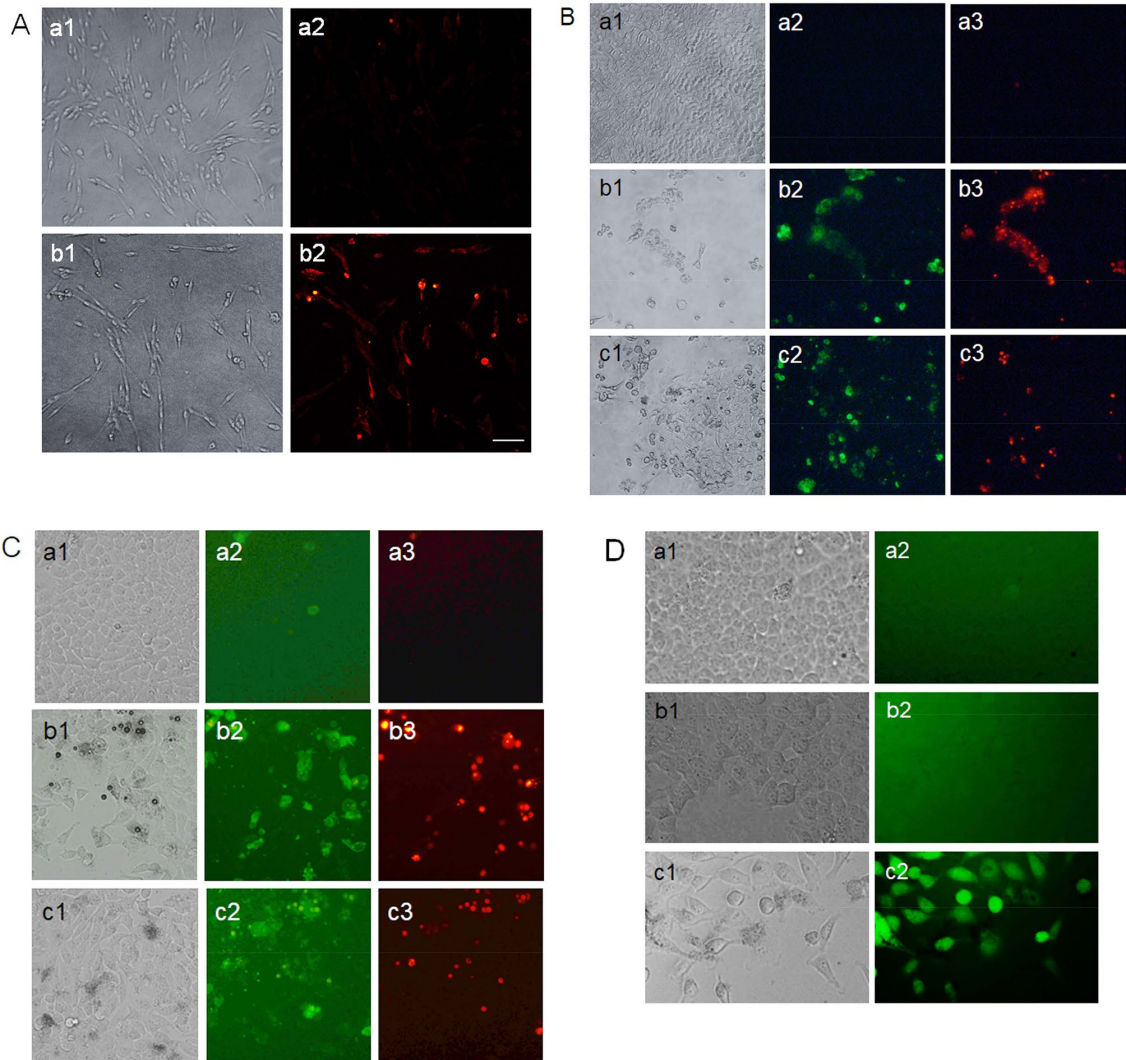
Cell morphological studies by the fluorometric assays. (**A**) Detection of apoptosis in GSCs by immunofluorometric assay. Where a1 and b1 represent the optical microscopic images of the untreated and *G. densiflorum*-Ag/AgCl-NPs treated GSCs, respectively; a2 and b2 represent the fluorescence microscopic images of the untreated and *G. densiflorum*-Ag/AgCl-NPs treated GSCs, respectively after staining with Cy3 goat-anti Rabbit IgG antibody. (**B**) Detection of early and late apoptosis in BxPC-3 by FITC-annexin V/PI. (**C**) Detection of early and late apoptosis in MCF-7 cells by FITC-annexin V/PI. In the Figures (**B**) and (**C**), a1, b1 and c1 indicate the optical microscopic images of untreated, *G. densiflorum*-Ag/AgCl-NPs and *K. rotunda*-Ag/AgCl-NPs treated cells, respectively; a2, b2 and c2 indicate the fluorescence microscopic images of untreated, *G. densiflorum*-Ag/AgCl-NPs and *K. rotunda*-Ag/AgCl-NPs treated cells, respectively after stained with FITC-annexin V; a3, b3, and c3 indicate the fluorescence microscopic images of untreated, *G. densiflorum*-Ag/AgCl-NPs and *K. rotunda*-Ag/AgCl-NPs, treated cells, respectively after stained with PI. (**D**) ROS generation in MCF-7 cells. Where a1, b1 and c1 represent the optical microscopic images of the untreated control, *G. densiflorum*-Ag/AgCl-NPs and *K. rotunda*-Ag/AgCl-NPs treated MCF-7 cells, respectively; a2, b2 and c2 represent the optical microscopic images of the untreated control, *G. densiflorum*-Ag/AgCl-NPs and *K. rotunda*-Ag/AgCl-NPs treated MCF-7 cells, respectively after stained with DCFH-DA. All image was captured at 20 × magnification. In Fig (**A**), the solid bar indicates 20 m.

After incubation of GSCs with the *G. densiflorum*-Ag/AgCl-NPs, the active caspase-3 protein expressions were increased remarkably as presented in Fig. 4A. Early and late apoptotic cells in the microscopic image were detected after treatment of BxPC-3 and MCF-7 cells with the *G. densiflorum-*Ag/AgCl-NPs and *K. rotunda-*Ag/AgCl-NPs as illustrated in Fig. 4B and C, respectively. ROS was generated in MCF-7 cells after treatment with *K. rotunda-*Ag/AgCl-NPs, while no ROS was detected in control and *G. densiflorum*-Ag/AgCl-NPs (Fig. 4D).

### Changes of gene expression in the treated GSCs and MCF-7 cells

**Figure 5:**
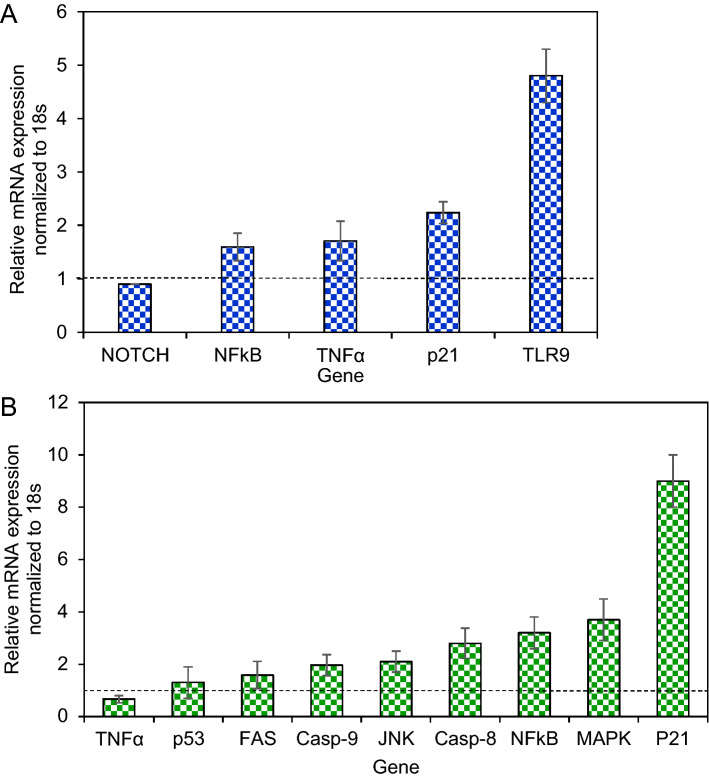
Assessment of genes expression in GSCs and MCF-7 cells. After treatment of GSCs (**A**) and MCF-7 (**B**) with *G. densiflorum*-Ag/AgCl-NPs, changes of genes expiration were analyzed by the Double Delta CT method. The data were normalized using the 18 s gene. A dashed line (black) indicates 1.0 expression level.

After incubation of GSCs with the *G. densiflorum*-Ag/AgCl-NPs, the expression level of *NFκB, TNFα, p21* and *TLR9* increased while almost no change was observed in the *NOTCH2* gene (Fig. 5A). The expression level of *caspase-8, NFκB, MAPK* and *p21* were increased significantly while *p53, caspase-9,* and *FAS* expression increased a little and no big alteration was found for *TNFα*, in MCF-7 after treatment with *G. densiflorum*-Ag/AgCl-NPs (Fig. 5B).

### EAC cells growth inhibition and hematological parameters

**Figure 6:**
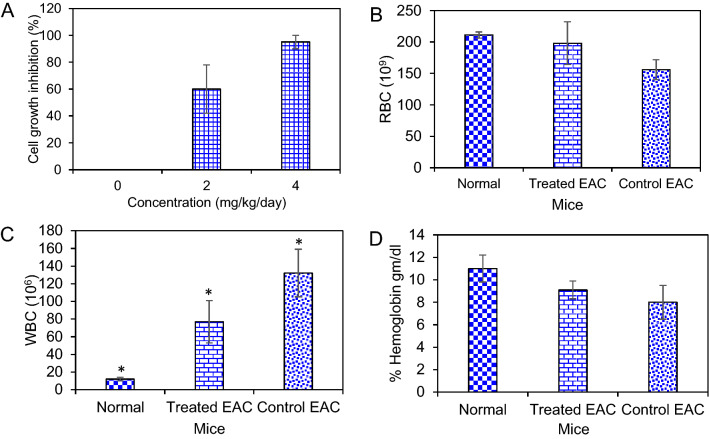
Cells growth inhibition and hematological parameter. (**A**) The number of EAC cells was counted in EAC-control and *G. densiflorum*-Ag/AgCl-NPs treated EAC-bearing mice. (**B**), (**C**) and (**D**) indicate the changes in RBC, WBC and % of hemoglobin after treatment with the nanoparticles. *P < 0.05.

After treatment with *G. densiflorum*-Ag/AgCl-NPs, EAC cells growth was inhibited effectively. At the dose of 2 mg/Kg/day, cell growth inhibition was 60% and the growth inhibition increased to 95% at the dose of 4 mg/Kg/day as shown in Fig. 6A. A clear difference in hematological parameters was observed between normal, tumor-bearing and *G. densiflorum*-Ag/AgCl-NPs treated mice. Total RBC of normal and *G. densiflorum*-Ag/AgCl-NPs treated EAC bearing mice were very close when compared with the EAC bearing control mice (Fig. 6B). A remarkable decrease in WBC was observed after treatment of EAC-bearing mice with *G. densiflorum*-Ag/AgCl-NPs (Fig. 6C). The Hemoglobin level increased after treatment of EAC-bearing mice with the *G. densiflorum*-Ag/AgCl-NPs (6D).

### Effects of *G. densiflorum*-Ag/AgCl-NPs on average tumor growth and the mean survival time of EAC Cells bearing mice

**Figure 7:**
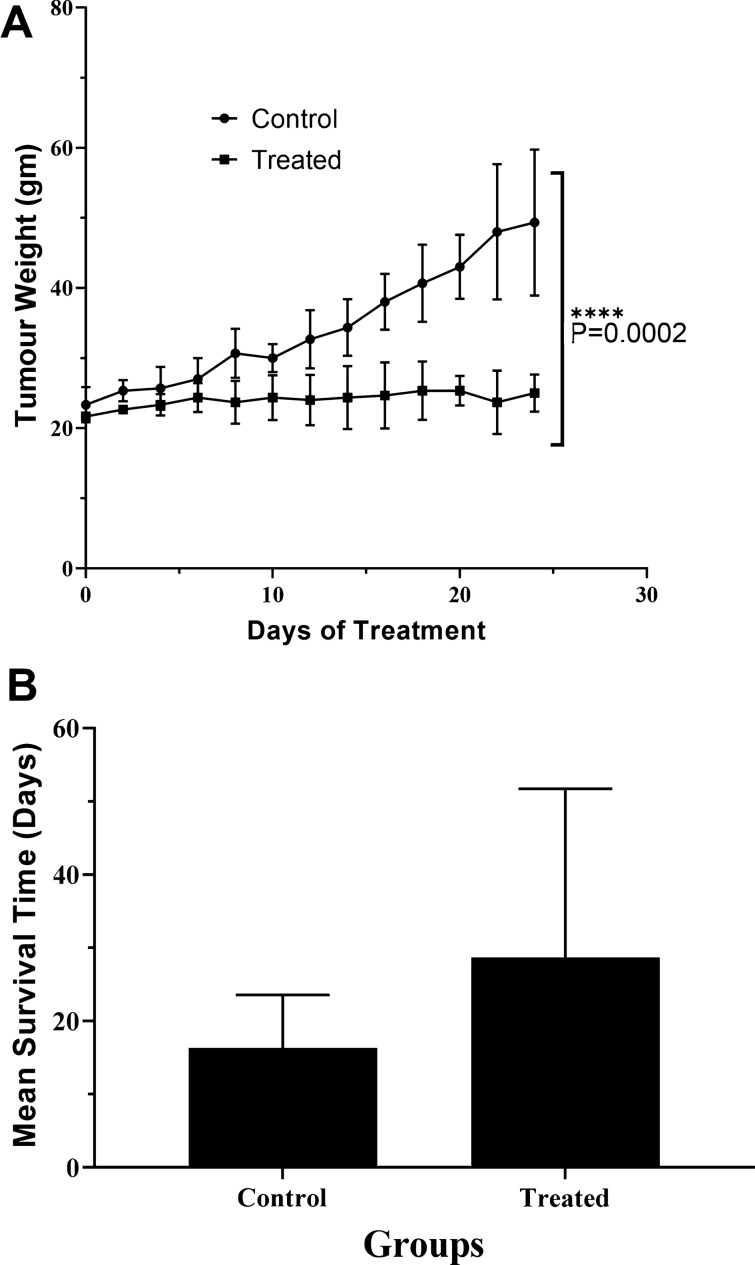
Average tumor weight and life span. Changes of average tumor weight (**A**) and increase in life span (**B**) after treatment with the *G. densiflorum*-Ag/AgCl-NPs.

EAC-bear mice were treated with *G. densiflorum*-Ag/AgCl-NPs and 20 days later, the average tumor growth in mice was remarkably low as compared with the EAC-bearing control mice (Fig. 7A). The life span of the *G. densiflorum*-Ag/AgCl-NPs treated mice was increased by 75% as compared to EAC-bearing control mice (Fig. 7B).

## Discussion

For the treatment of cancer, scientists are finding different therapeutic agents those stimulates apoptosis in the cancer cell. The apoptotic inducers derived from phytochemicals have attracted much attention to researchers due to the less toxic effects than those of radiation and chemically synthesized chemotherapeutic agents. Synthesis of nanoparticles by treating AgNO_3_ with such type of extract attracted several cancer researchers. For this reason, we have synthesized some nanoparticles which showed cytotoxicity against different cancer cell lines^1,2,17^. In the present study *G. densiflorum* rhizome was used for the first time for the synthesis of Ag/AgCl-NPs by treating with AgNO_3._ After synthesis of Ag/AgCl-NPs the color of the extract was changed from transparent to deep brown and showed a maximum absorbance peak around 455 nm due to the surface plasmon resonance^25^. The spherical size nanoparticles with an average size of 25 nm were confirmed by SEM. In our past study, spherical Ag/AgCl-NPs were also obtained from the *K. rotunda* rhizome and *Z. mauritiana* fruit extracts with an average size of 17 and 16 nm, respectively^1,2^. Several studies revealed that most of the synthesized biogenic Ag/AgCl-NPs are spherical^6^. The XRD data confirmed the crystal-like nature of the *G. densiflorum*-Ag/AgCl-NPs, which contained silver and silver chloride. Ag/AgCl-NPs were also synthesized from the *K. rotunda* rhizome and *Z. mauritiana* fruit extract and the possible source of chlorine for the formation of AgCl-NPs is Tris–HCl which was discussed in our early investigation^1,2^. Investigation of the heat stability of the *G. densiflorum*-Ag/AgCl-NPs showed that the nanoparticles lost their weight in three different stages as reported earlier for *K. rotunda-*Ag/AgCl-NPs and *Z. mauritiana*-Ag/AgCl-NPs^1,2^. Removal of the water from the nanoparticles was the cause of the first weight loss, whereas decomposition or elimination of capping biomolecules would be the reason for the second and third weight loss of the nanoparticles^26^. It indicates the high thermal stability of the *G. densiflorum*-Ag/AgCl-NPs. The FTIR frequencies represent the bonds of –OH, –CH, N–H, N–O, and C–Cl stretches that indicate the presence of alcohol/phenol, alkanes, primary amines, nitro compounds, and alkyl chloride functional groups in the *G. densiflorum*-Ag/AgCl-NPs.

Several studies showed that biogenic silver nanoparticles retain the ability to inhibit cancer cell growth in vitro and also in vivo in mice and the ability to inhibit cancer cell growth varies on the source of the synthesized silver nanoparticles^1,2,19,27–32^. In the present investigation, we have examined the anticancer activity of the *G. densiflorum*-Ag/AgCl-NPs, *K. rotunda*-Ag/AgCl-NPs and *Z. mauritiana*-Ag/AgCl-NPs against BxPC-3 cells for the first time for any biogenic silver nanoparticles. The IC_50_ values indicated *G. densiflorum*-Ag/AgCl-NPs were the most toxic against BxPC-3 cells. Then the mechanism of anticancer activity of *G. densiflorum*-Ag/AgCl-NPs and *K. rotunda*-Ag/AgCl-NPs against BxPC-3 was studied using FITC-annexin V/PI assay. Both of the nanoparticles inhibited cell growth by the induction of early and late apoptosis.

**Figure 8:**
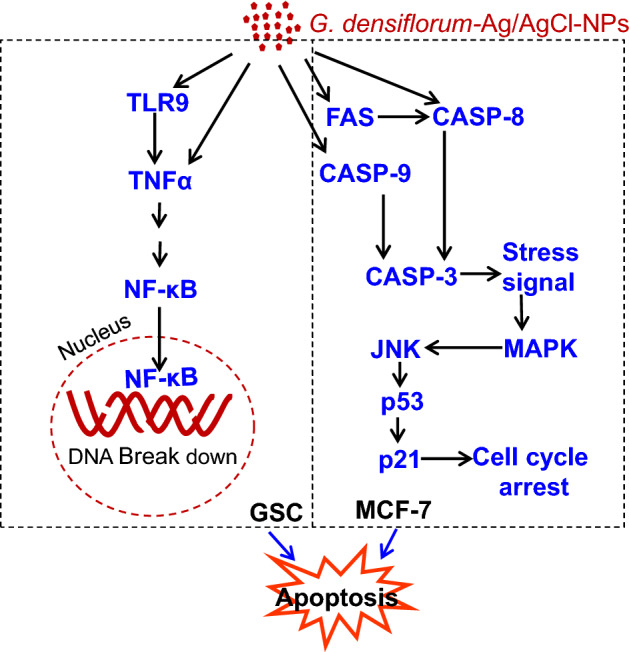
Schematic representation of the effects of *G. densiflorum*-Ag/AgCl-NPs on the induction of apoptosis in GSCs and MCF-7 cells.

Anticancer activity of the *G. densiflorum*-Ag/AgCl-NPs was also checked against GSCs cells and inhibition of the growth of the cells was observed with the IC_50_ value of 28.0 µg/ml. Recently, it was reported that *K. rotunda*-Ag/AgCl-NPs and *Z. mauritiana*-Ag/AgCl-NPs also inhibited the GSCs with the IC_50_ values of 6.8 and 10.4 μg/ml^1^. That demonstrated the newly synthesized nanoparticles were less effective against GSCs. Cell growth inhibition was due to the induction of apoptosis which was confirmed by the immunofluorescence study of caspase-3 protein expressions. Similar results were also obtained in our early experiments of the *K. rotunda*-Ag/AgCl-NPs and *Z. mauritiana*-Ag/AgCl-NPs against GSCs^1^. Here the expression level of *TLR9, p21, TNFα* and *NFκB* genes increased and almost no change was observed for *NOTCH2* gene that is responsible for self-renewal, cell proliferation and cell maintenance of GSCs. Our previous study demonstrated that *K. rotunda*-Ag/AgCl-NPs inhibited the GSCs by increasing the *TLR9, p21, TNFα* and *NFκB* genes with the several folds decrease of *NOTCH2*^1^. Like our early report, the present study also illustrated that *G. densiflorum-*Ag/AgCl-NPs activated *TLR9* gene that activated NFκB sequentially by activating TNFα and, finally, NFκB entered the nucleus and broke the DNA, resulting in induction of apoptosis in GSCs cells. Opposite result was also reported by Akter et al.^33^. They found *Brassica rapa* var. japonica (Bj) leaf extract mediated silver nanoparticles induced apoptosis in Caco-2 cells by decreasing the expression of NFκB protein. Besides the GSCs and BxPC-3 cells, *G. densiflorum*-Ag/AgCl-NPs and *K. rotunda*-Ag/AgCl-NPs also inhibited the growth of MCF-7 cells with the IC_50_ values of 21.5 and 23.5 μg/ml. In our previous study, it was found that *Z. mauritiana*-Ag/AgCl-NPs and *H. musciformis*-Ag/AgCl-NPs inhibited MCF-7 cells with the IC_50_ value of 28 and 36.95 μg/ml and induced apoptosis^2,17^. In the present study, *G. densiflorum*-Ag/AgCl-NPs and *K. rotunda*-Ag/AgCl-NPs caused cell death by inducing early and late apoptosis in the MCF-7 cells. Different cellular processes e.g., ROS generation, death-induced signals and caspase up-regulation, are related to apoptosis. In several studies, it was found that cellular uptake of AgNPs leads to the generation of ROS in MCF-7 cells, which causes oxidative stress in the cells and apoptosis induced in the cells^2^. Using DCFH-DA dye, ROS generation was observed in this study. Like, *Z. mauritiana*-Ag/AgCl-NPs^2^, *K. rotunda*-Ag/AgCl-NPs produced ROS in the MCF-7 cells while no ROS generation was observed after treatment with *G. densiflorum*-Ag/AgCl-NPs and *H. musciformis*-Ag/AgCl-NPs. *Z. mauritiana*-Ag/AgCl-NPs, caused increased expressions of *caspase-8, FADD, FAS* genes with the decreased *PARP* gene in MCF-7 cell^2^ while *G. densiflorum*-Ag/AgCl-NPs caused a remarkable increase of the expressions of *p21, MAPK, NFκB* and *caspase-8* genes with the observable increase of *JNK, Casp-9, FAS, p53* genes and little decreased of *TNFα* gene in this experiment. NFκB is a well-known gene required for cell growth and survival. The gene can work as a pro-apoptotic or anti-apoptotic regulatory factor and the type of function is regulated by the nature of the apoptotic stimulus^34,35^. In the present study, the nanoparticles stimulated the gene as a pro-apoptotic regulatory factor that may help to induce apoptosis in MCF-7 cells. Increased expression of *FAS* and *caspase-8* genes indicated the possibility of the involvement of FAS pathways. The expression of the *capsase-9* gene indicates the possibility of the involvement of the intrinsic pathway. MAPK can act as an activator or inhibitor of apoptosis that depends on the type of cells and nature of the stimulus^36^ and JNK riserises the expressions of several pro-apoptotic proteins by regulating the different transcription factors^37,38^. In the MAPK-JNK signaling pathway, JNK is stimulated by MAPK and p53 is activated by JNK. p53 is a well-known gene that causes apoptosis in cells in several ways. Here, increased expression of the *p53* gene may be activated the *p21* gene that caused cell cycle arrest^1^. Effects of the *G. densiflorum*-Ag/AgCl-NPs on the GSCs and MCF-7 cells were pictorially represented in Fig. 8. Antiproliferative activity of different Ag-NPs against the MCF-7 cells was reported from different laboratories^27,39–42^ but anticancer mechanisms were studied in only a few cases^43–46^. Baharara et al. reported that *Achillea biebersteinii*-AgNPs induced apoptosis in the MCF-7^43^. Where the nanoparticles downregulated the expression of the Bcl-2 gene and upregulated the Bax gene and activated caspase-3 &-9. Fard et al. reported that *Centella asiatica*-AgNPs inhibited MCF-7 also by induction of apoptosis and up-regulation of *caspase-3* &-*9* genes^44^. Gandhi et al. Ps-AgNPs induced apoptosis in MCF-7 cells the downregulation of inflammatory genes (TNF-alpha and IL-6) and cell cycle genes (PCNA and Cyclin-D1)^46^. Our present study stated the involvement of various genes in the apoptosis process of the MCF-7.

In vitro anticancer study using biogenic Ag/AgCl-NPs is common but only view literature stated the in vivo study^9,12–14^. In our previous study, we found that *Z mauritiana-*Ag/AgCl-NPs and *K rotunda-*Ag/AgCl-NPs inhibited 20% and 55% of EAC cells growth inhibition at the dose of 12 mg/Kg/day dose, respectively. While the dose was decreased to 6 mg/Kg/day no cell growth was observed for *Z mauritiana-*Ag/AgCl-NPs and 32.3% of EAC cells growth inhibition was observed for *K rotunda-*Ag/AgCl-NPs^1,2^. However, in the present study, 95% and 60% of cells growth inhibition were observed at 4 and 2 mg/Kg/day doses, respectively. The result indicated the *G. densiflorum*-Ag/AgCl-NPs is more active than that *Z mauritiana-*Ag/AgCl-NPs and *K rotunda-*Ag/AgCl-NPs.

Besides the EAC cells growth inhibition, for the first time, we are reporting the result of the investigation of the hematological parameters, average tumor growth and life span. After treatment of biogenic silver nanoparticles, the total RBC and hemoglobin level in the *G. densiflorum*-Ag/AgCl-NPs treated EAC bearing mice increased with the decrease of WBC. Typically, anemia occurs in the EAC-bearing mice due to the decrease of RBC and percent of hemoglobin. After treatment with the nanoparticles, RBC and hemoglobin levels were increased and the infection was reduced as the WBC was decreased. In the EAC-bearing mice, a rapid increase of ascites fluid is observed. The ascites fluid is the direct nutritional source of EAC cells. After treatment with *G. densiflorum*-Ag/AgCl-NPs, the volume of ascites fluid and the number of EAC cells decreased with the increased life span. These results indicated the synthesized nanoparticles would be a candidate for strong anticancer agents.

## Conclusion

In conclusion, *G. densiflorum*-Ag/AgCl-NPs were synthesized and characterized that retained the anticancer activity against BxPC-3, GSCs and MCF-7 cells. Although the anticancer activity of the *G. densiflorum*-Ag/AgCl-NPs against BxPC-3 and MCF-7 was stronger than *K. rotunda*-Ag/AgCl-NPs and *Z. mauritiana*-Ag/AgCl-NPs, however, weaker than those nanoparticles against GSCs. The *G. densiflorum*-Ag/AgCl-NPs might be a strong anticancer agent against the BxPC-3 and MCF-7 cells. In vivo experiments indicated that *G. densiflorum*-Ag/AgCl-NPs can be a promising anticancer agent and can be used for further investigations.

### Statistical analysis

The experimental results have been expressed as the mean ± SD (Standard Deviation). Data have been calculated by the one-way ANOVA followed by the Dunnett ‘t’ test using SPSS software of version 16.
